# Frequency-dependent selection by wild birds promotes polymorphism in model salamanders

**DOI:** 10.1186/1472-6785-9-12

**Published:** 2009-05-08

**Authors:** Benjamin M Fitzpatrick, Kim Shook, Reuben Izally

**Affiliations:** 1 Ecology & Evolutionary Biology, University of Tennessee, Knoxville TN 37996, USA; 2 Pre-collegiate Research Scholars Program, University of Tennessee, Knoxville TN 37996, USA; 3 Farragut High School, Knoxville TN 37934, USA

## Abstract

**Background:**

Co-occurrence of distinct colour forms is a classic paradox in evolutionary ecology because both selection and drift tend to remove variation from populations. Apostatic selection, the primary hypothesis for maintenance of colour polymorphism in cryptic animals, proposes that visual predators focus on common forms of prey, resulting in higher survival of rare forms. Empirical tests of this frequency-dependent foraging hypothesis are rare, and the link between predator behaviour and maintenance of variation in prey has been difficult to confirm. Here, we show that predatory birds can act as agents of frequency-dependent selection on terrestrial salamanders. Polymorphism for presence/absence of a dorsal stripe is widespread in many salamander species and its maintenance is a long-standing mystery.

**Results:**

We used realistic food-bearing model salamanders to test whether selection by wild birds maintains a stripe/no-stripe polymorphism. In experimental manipulations, whichever form was most common was most likely to be attacked by ground-foraging birds, resulting in a survival advantage for the rare form.

**Conclusion:**

This experiment demonstrates that frequency-dependent foraging by wild birds can maintain colour polymorphism in cryptic prey.

## Background

Maintenance of variation is a classic paradox in evolution because both selection and drift tend to remove variation from populations [1-3]. If one form has an advantage (e.g., being more cryptic), it should replace all others. Likewise, random drift alone will eventually result in loss of all but one form when there are no fitness differences. Maintenance of a stable polymorphism requires either recurrent mutation, a balance between dispersal and divergent selection between populations, or some form of balancing selection within populations [3]. For example, predators are expected to form a search image for the most common form if it is easier to search for one cryptic prey type than to simultaneously look for two [4-6]. If predators switch their search images to whichever prey is most common, the result is frequency-dependent selection against common prey [4-7]. Empirical tests of this frequency-dependent selection hypothesis are rare, and the link between predator behaviour and maintenance of variation in prey has been difficult to confirm [4,8]. Here, we test whether predatory birds can act as agents of frequency-dependent selection on terrestrial salamanders using a manipulative field experiment.

**Figure 1:**
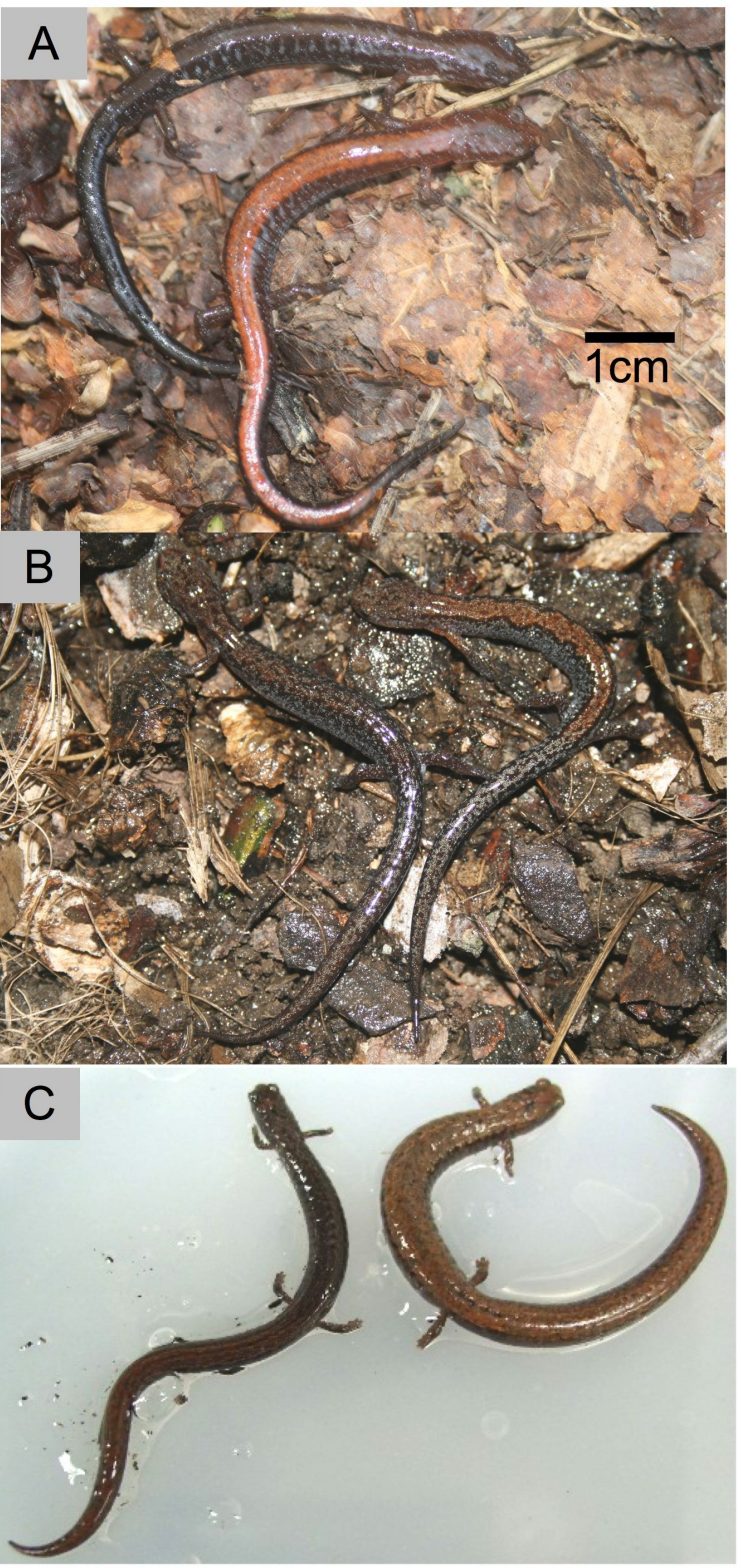
**Examples of polymorphic plethodontid salamanders**. Each pair of individuals was captured in the same place at the same time. a, Southern Red-backed Salamanders, *Plethodon serratus*, Great Smoky Mountains National Park (Tennessee, USA). b, Southern Zigzag Salamanders, *P. ventralis*, Knoxville (Tennessee, USA). c, California Slender Salamanders, *Batrachoseps attenuatus*, Napa County (California, USA).

Multiple species of small, forest floor-dwelling salamanders exhibit polymorphism for the presence of a dorsal stripe (Fig. 1) and what maintains this polymorphism within populations is a long-standing question [9-15]. Polymorphism is widespread both geographically and phylogenetically, occurring in many species of *Plethodon *throughout North America in addition to the distantly related *Batrachoseps *of the Pacific coast of North America [16] and *Karsenia *of Asia [17]. Thus, it is unlikely that this polymorphism is merely a transitional stage in the evolution of colour pattern. Presence or absence of a dorsal stripe appears to have a simple genetic basis in *Plethodon cinereus *and is not related to sex [12,18].

What polymorphic salamander species seem to have in common is that they are small, slender, and locally abundant. These species often comprise a substantial fraction of the animal biomass in temperate forests and are subject to predation by ground-foraging birds and other predators that search the leaf-litter for small animals [16]. Studies of polymorphic *Plethodon cinereus *have shown differences between the morphs in behaviour [11,14,15,19], physiology [11,13], and geographic variation in relative abundance [10,20]. However, none directly address how these differences might relate to a mechanism maintaining variation and there is no evidence of similar phenotypic correlations in the other polymorphic species. In fact, in *P. ventralis*, the frequency of the striped form is less at higher elevations [21] whereas the frequency of striped *P. cinereus *is greater at higher latitude and cooler microclimates in general [10,20]. Thus, phenotypic correlations between appearance and physiology are not consistent (also see [13]) and this is not surprising given that dorsal colour pattern is unlikely to have a direct function in temperature tolerance or metabolism (*Plethodon *always avoid sunlight).

To explain the maintenance of a similar visual polymorphism across many taxa, we propose that selection acts directly on appearance. The most likely function of dorsal colour pattern is crypsis (Fig. 1), though this is an untested assumption. Though there has been speculation that the red stripe of *P. cinereus *might be an aposematic signal [19], Brodie and Brodie [22] showed that wild birds prey extensively upon striped *P. cinereus*, and give no sign that the salamanders are unpalatable. Striped *P. cinereus *were taken just as often as *D. ochrophaeus*, which were assumed to be cryptic and palatable [22]. A third, completely red form of *P. cinereus *does appear to be a colour mimic of the toxic Red-spotted Newt eft (*Notophthalmus viridescens*), but our focus is on the more widespread stripe/no-stripe polymorphism. Polymorphism in cryptic prey populations might be maintained in migration-selection balance if alternative colour forms are adapted to different habitats [23], or might be promoted by frequency-dependent predation causing rare form advantage within populations [4,8].

Empirical tests of frequency-dependent foraging on cryptic prey are rare; the best-studied examples involve either colored pellets of dough fed to free-ranging birds [7,24,25]or digital images of moths selected by trained Blue Jays (*Cyanocitta cristata*) [4,26-28]. These studies demonstrate that birds can generate frequency-dependent selection, that individual Blue Jays learn to focus on abundant prey, and that they readily switch focus in response to changes in prey abundance. Apostatic selection by free-ranging wild birds has been shown for small pastries differing in colour [7,25,29], and presence or absence of a stripe [30,31], and also for real *Cepaea *snail shells differing in banding pattern [32]. However, in other studies, selection changed from negative frequency-dependence (rare form advantage) to positive frequency-dependence (common form advantage) when artificial prey were provided at high densities, showing that rare form advantage is not entirely general and may depend on abundance or aggregation of prey [25,33]. A recent study of Trinidadian guppies implicated frequency-dependent survival in the maintenance of variation in conspicuously colored males [34]. However, it is not clear whether search images are important for predators of conspicuous prey, and other factors might affect polymorphism in sexually-selected traits [35,36]. Thus, whether frequency-dependent foraging can explain the maintenance of polymorphism in realistic prey at realistic densities remains a critical question that might need to be addressed on a case-by-case basis. Here, we show empirically that predatory birds can act as agents of frequency-dependent selection on terrestrial salamanders.

**Figure 2:**
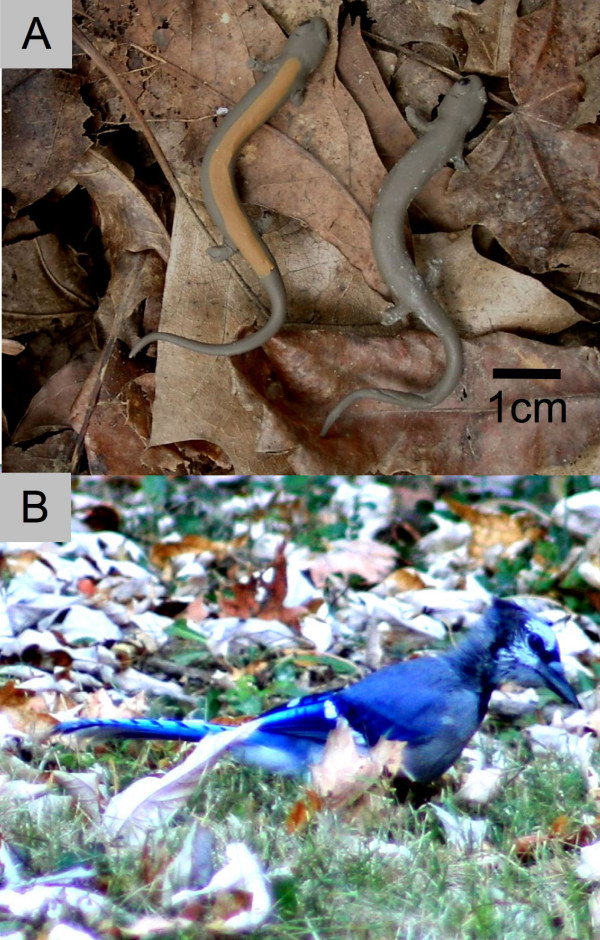
**Experimental subjects**. (a) Polymorphic model salamanders. (b) Blue Jay (*Cyanocitta cristata*) foraging in the study area.

To test for frequency-dependent selection by birds, we used standardized models resembling polymorphic *Plethodon *salamanders (Fig. 2). A food reward (1/2 peanut) was glued to the underside of each model, and models were set out at random in 10 × 10 m plot at the edge of a woodlot in Knox county, Tennessee (see Methods). We manipulated the relative abundance of striped and unstriped prey and quantified survival by counting the number of models with peanuts still attached at the end of each day.

## Results and Discussion

We observed at least five individual Blue Jays (*Cyanocitta cristata*) foraging on model salamanders. Other birds observed foraging on the ground in the study site included Northern Cardinals (*Cardinalis cardinalis*), American Crows (*Corvus brachyrhynchos*), American Robins (*Turdus migratorius*), and Eastern Towhees (*Pipilio erythrophthalmus*). Only Blue Jays were observed taking peanuts from salamanders.

On the first test day, after unstriped models had been more abundant (9:1 for six days), more unstriped than striped model salamanders were attacked (day 7: 16/25 vs. 6/25, Fisher's exact *P *= 0.0096). After six days with 5 unstriped and 45 striped models, fewer unstriped models were attacked when tested at equal abundance (day 14: 11/25 vs. 19/25, Fisher's exact *P *= 0.0421). Whichever form had been rare (1:9) over the past six days had a significant survival advantage over the common form when presented at equal abundance (Table 1).

**Table 1:** "Survival" rates of model salamanders. Data are given as number survived/number presented each day.

Date^a^	Stripe	No Stripe
6 Jul	1/5	17/45
7 Jul	4/5	21/45
8 Jul	4/5	20/45
9 Jul	4/5	30/45
10 Jul	5/5	35/45
11 Jul	4/5	24/45
12 Jul	19/25	9/25
13 Jul	35/45	3/5
14 Jul	25/45	3/5
15 Jul	16/45	3/5
16 Jul	26/45	4/5
17 Jul	26/45	4/5
18 Jul	25/45	4/5
19 Jul	6/25	14/25

^a ^2008 AD

**Figure 3:**
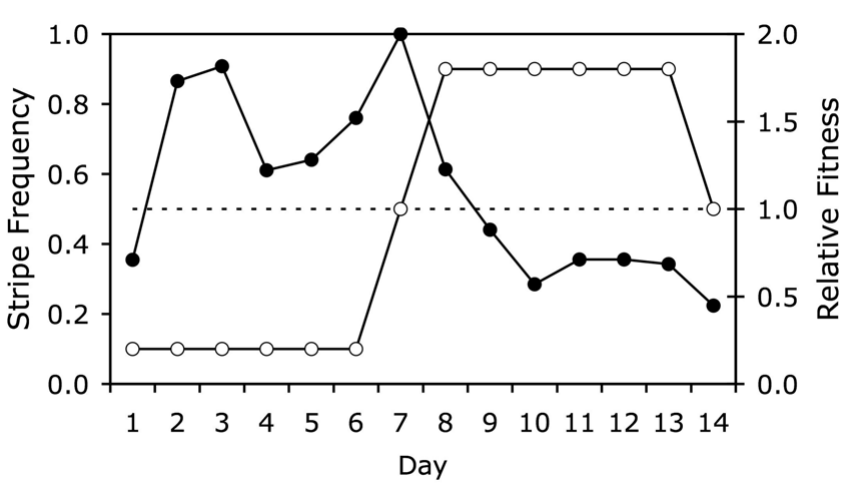
**Relative fitness of model salamanders**. Daily survival rates of striped relative to unstriped models (right-hand axis, filled symbols) in comparison to the frequency of striped models (left-hand axis, open symbols). Dashed line illustrates equal frequency and equal fitness.

Fig. 3 shows that striped models had higher relative fitness when at low frequency and lower relative fitness when at high frequency. Multiple logistic regression supported a significant relationship between frequency and survival on non-test days (likelihood ratio *G*^2 ^= 6.37, df = 1, *P *= 0.0116). There was also a strong effect of "day" (*G*^2 ^= 58.27, df = 12, *P *< 0.0001), which probably reflects variation in predator activity (predation was lighter on rainy days) and a delay in learning. There was no detectable association between survival and morph (likelihood ratio *G*^2 ^= 0.225, df = 1, *P *= 0.635). Whichever form was rare had a survival advantage within one or two days after the relative abundances were reversed (Fig. 3). Thus, when wild birds were "trained" to expect one form to be most common, they preyed disproportionately on that form. This occurred regardless of which form was common.

Selection by birds can be heterogeneous and these results should be tested at other sites. However, decades of research have consistently found that captive and free-ranging wild birds cause frequency-dependent selection on all manner of food items differing in appearance [4,6,7,25,26,28]. We show that this very general process can operate on the kind of phenotypic polymorphism seen commonly in small salamanders.

We have not tested the perceptual and behavioural mechanisms underlying frequency-dependent foraging, therefore we have avoided invoking search image formation as the specific ultimate cause of rare form advantage. Nevertheless, this study joins a growing body of literature showing that birds often switch foraging behaviour in response to the relative abundance of alternative cryptic prey and this can promote the maintenance of colour polymorphism [4,8]. The cognitive processes underlying this phenomenon are important subjects for further research.

In addition, a crucial question for the maintenance of polymorphism is to what extent frequency-dependent foraging by visual predators is counteracted by other selective processes, including non-visual predation. Other sources of mortality and other phenotypic differences between morphs might interfere with or enhance the stable coexistence of distinct colour morphs. The conceptual link between promoting polymorphism and promoting coexistence of similar species [37] merits greater attention.

## Conclusion

Rare form advantage owing to frequency-dependent selection by visual predators has been proposed to explain maintenance of polymorphism in many cryptic species [4]. The experiment reported here provides evidence that the key mechanism (frequency-dependent foraging) operates with realistic prey at realistic densities in the wild. The maintenance of colour variation in terrestrial salamanders might be explained by the oldest and most obvious hypothesis – rare form advantage arises because predators tend to overlook rare prey.

## Methods

Model salamanders were constructed with modelling clay (Sculpey™). One model was sculpted by hand and used to create a flexible mould. This mould was then used to make models of consistent size and body position. Models were either grey-brown or grey-brown with an ochre stripe from the base of the head to the base of the tail (Fig. 2a). A food reward was associated with each model salamander by adhering one peanut half to the bottom of the model using edible library paste (40 g corn starch, 1 L water, boiled and cooled).

The field site was an open area adjacent to a woodlot in Knox County, TN. Polymorphic *P. ventralis *are known from within 2 km. To avoid spurious loss of models, we used as foraging units 100 black plastic trays (0.185 m^2^) placed in a 10 × 10 array and filled with partially decomposed leaf-litter (natural forest floor substrate). One m of open lawn was left between adjacent trays. Each day, we used a random number generator to assign model salamanders to 50 trays. The ordering of striped and unstriped models was also random; as a result, each tray position had a 50% chance of having a model salamander each day and the probability that the model was striped was the frequency of striped models for that day. The resulting density of 1/2 m^-2 ^was realistic for small *Plethodon *and *Batrachoseps *salamanders [16]. Models were placed haphazardly within the trays and arranged so that the attached peanut could not be seen without moving the model. This design ensured that foraging birds had the opportunity to learn where to look for salamanders and that all "surviving" salamanders would be recovered at the end of each day. In addition, it was important to make sure that each tray was not guaranteed to provide a salamander, so that sometimes giving up and moving to another was the right foraging decision [27].

The experiment was divided into two training periods of six days each and two test days, similar to protocols used with green and brown pastry pellets[7,25]. First, 5 striped and 45 unstriped salamander models were presented each day from 8:00 am until 7:30 pm (it usually took this entire time for the birds to attack roughly half of the salamander models). At the end of the day, the flats were gathered and the number of "surviving" salamanders counted (those with the peanut still attached). Six days of training were followed immediately by a test day in which 25 of each morph were presented. After the first test day, another six-day training period was started, this time with frequencies of 45 striped and 5 unstriped salamander models. The second test day (25 of each morph) immediately followed the second training period (Table 1).

We used 2 × 2 contingency tables to test the null hypothesis of equal predation rates for striped and unstriped models on the two test days. For the 12 "training" days, we tested for an effect of relative abundance on survival using logistic regression with individual survival as the response variable, day as a random effect, and morph and frequency category as fixed effects. The goodness-of-fit of the model was adequate (likelihood ratio test relative to a saturated model: *G*^2 ^= 4.03, df = 11, *P *= 0.708) and adding interaction terms did not significantly improve the fit. For all 14 days of the experiment, we estimated fitness (survival value) of striped relative to unstriped models as *w*_*S *_= [(*S*_*A *_+ 0.5)/(*S*_*B *_+ 0.5)] ÷ [(*U*_*A *_+ 0.5)/(*U*_*B *_+ 0.5)], where *S*_*A *_and *U*_*A *_are the numbers of surviving striped and unstriped models, and *S*_*B *_and *U*_*B *_are the numbers before selection. This estimates the ratio of the survival probability of striped relative to unstriped models [38]. For example, when the two forms have equal fitness, *w*_*S *_= 1.0 and when a striped individual is twice as likely to survive as an unstriped individual, *w*_*S *_= 2.0.

## Authors' contributions

BMF Conceived the project, collected and analyzed the data, and drafted the manuscript. KS and RI helped troubleshoot the experimental design, collect and analyze data, and revise the manuscript. All authors read and approved the final manuscript.
